# Integrating Continuous and Batch Processes with Shared Resources in Closed-Loop Scheduling: A Case Study on Tuna Cannery

**DOI:** 10.1021/acs.iecr.3c00754

**Published:** 2023-06-02

**Authors:** Carlos G. Palacín, José L. Pitarch, Carlos Vilas, César de Prada

**Affiliations:** † Industrial Digital Transformation, Sonae Arauco SA, Valladolid 47009, Spain; ‡ Control of Complex Systems, Instituto de Automática e Informática Industrial (ai2), Universitat Politècnica de València, Valencia 46022, Spain; § Biosystems and Bioprocess Engineering, Institute for Marine Research (IIM), CSIC, Vigo 36208, Spain; ∥ Process Supervision and Control, Systems Engineering and Automatic Control Dept. & Institute of Sustainable Processes (ISP), Universidad de Valladolid, Valladolid 47011, Spain

## Abstract

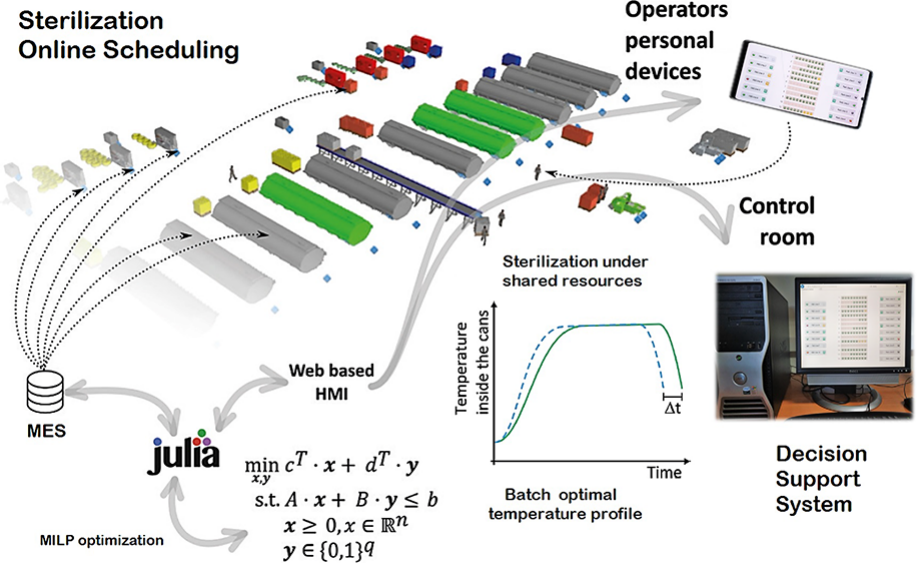

Scheduling tasks in production facilities are usually hybrid optimization problems of a large combinatorial nature. They involve solving, in near-real time, the integration of the operation of several batch units of continuous dynamics with the discrete manufacture of items in processing lines. Moreover, one has to deal with uncertainty (process delays, unexpected stops) and the management of shared resources (energy, water, etc.) including decisions made by plant operators: still, some tasks in the scheduling layers are done manually. Manufacturing Execution Systems (MESs) are intended to support plant personnel at this level. However, there is still much work to do in terms of performing automatic scheduling, computed in real time, that guides managers to achieve an optimal operation of such complex cyber-physical systems. This work proposes a closed-loop approach to handle the uncertainty arising when facing the online scheduling of supply lines and parallel batch units. These units often share some resources, so effects due to concurrent resource consumption on the system dynamics are explicitly considered in the presented formulation. The proposed decision support system is tested onsite in a tuna cannery, to handle short-term online scheduling of sterilization processes that deal with limited steam, carts, and operators as shared resources.

## I Introduction and Motivation

The process industry comprises several sectors where raw material is subjected to continuous physical and/or chemical treatments, but in some of them, the material fluxes are discrete, e.g., food industry or consumer goods. In this context, suitable planning and scheduling of production lines, treatments, and equipment are needed to meet deadlines and prevent bottlenecks but also to improve productivity and to reduce material waste as well as resource consumption. At this point, it is worth stressing the importance of integrating the information gathered from the plant in real time with the scheduling and planning layers to react to unexpected issues and to allow fully exploiting the technological advances of Industry 4.0 for decision-making.^1,2^

It might be said that there are as many ad-hoc scheduling strategies as companies, but computer-based decision support systems (DSSs) are key in seeking optimal schedules instead of just feasible ones. These tools let the engineer append economic objectives, such as minimizing energy consumption, the total processing time, or maximizing product quality.^3^

Mathematical programming methods^4^ have been widely adopted to be the core of such DSS by their flexibility to model complex systems and the use of efficient optimization algorithms that evaluate hundreds of combinatorial alternatives in seconds. Hence, mathematical optimization is often the way to address large-scale scheduling problems in complex process plants.^5^ However, while open-loop optimization (no feedback from the plant is used to recompute the solution) is enough for planning, the existing uncertainty in the form of disruptions at the tactical and operational levels (unplanned delays, equipment failure, changing demand, etc.) poses issues that often demand a rolling-horizon scheduling solution as an optimal recovery strategy.^6^ Such closed-loop approaches recompute the schedule online (i.e., in short periods) to incorporate the actual plant state, following a schema analogous to model predictive control and to handle disruptions while reducing deviations with the reference production plan. The first formulations of closed-loop scheduling via mathematical programming appeared recently.^7,8^ Basically, any formulation that can be adapted to run in “real time” (i.e., can be solved within the required re-computation period) is suitable for closed-loop scheduling as long as it models the existing processing features (setups, utility constraints, etc.) of a particular application with enough accuracy. Note that, in addition to the advances in computational power and optimization solvers, important research has been conducted during the last years to improve the performance of scheduling DSS, both from the modeling and solution strategies.^9,10^

Indeed, as the speed to get a solution is key in closed-loop scheduling, artificial intelligence (AI) developments have been recently tailored to learn ‘optimal’ scheduling policies from data.^11,12^ The main advantage is that the learned policy models provide solutions extremely fast, as no numerical optimization is involved. However, some well-known drawbacks limit the use of such AI approaches in complex industrial scheduling: (a) guaranteeing state constraints is difficult; (b) the scale is limited to small/medium size problems, otherwise the action-state space is extremely large to approximate a value function fairly enough; (c) learning optimal policies under uncertainty requires having a detailed simulation model to create extensive virtual data from thousands of runs with different state-input situations; (d) any change in the actual system structure (e.g., a new constraint or product) requires generating new valid data and re-training the AI model. In fact, the authors of a related work^13^ report that mixed-integer linear programming (MILP) outperforms reinforcement learning (RL) in terms of optimality, despite having done policy training with about 450,000 simulations of the uncertain model. Note also that such an optimality gap was already reported in a problem of about a thousand decision variables and the industrial case study tackled in this paper has more than five thousand, as will be shown in the next sections. Therefore, MILP formulations are preferable as long as they can approximate the true problem with enough fidelity and can provide solutions fast enough for online implementations.

Shared resources such as steam, water, electricity, or manpower are often an extra cause of deviation, as the total consumption demanded by the plants/devices could surpass the resource availability, hence inducing delays or leading to infeasible scheduling situations. If the equipment resource demands are known in advance, the scheduling problem is generally approached in the literature by discretizing time and limiting the number of plants/equipment that can simultaneously operate at each instant (i.e., ensuring utility limits). It is well known that this strategy keeps the computational cost low and simplifies stock management.^14^ However, if resource consumption is not constant over time, the obtained solutions are conservative, which results in unnecessarily longer completion times, lower productivity, and limited manufacturing flexibility. In these cases, continuous process dynamics need to be integrated somehow into the scheduling formulation.^15,16^ However, processes often obey some nonlinear principles that would lead to complex non-convex optimization problems without optimality guarantees. While this limitation can be affordable in a real-time (static) optimization framework,^17^ it is not computationally viable for online scheduling problems. This is why much research is done in proposing efficient MILP formulations that approximate the reality well enough. In particular, the stage-precedence formulation is reported to be the preferred one in applications where a varying equipment performance (or resource consumption) over time is a key aspect.^18^ Nevertheless, to the authors’ knowledge, the closed-loop MILP scheduling proposals available in the literature do not incorporate the effect that concurrent resource consumption has over equipment dynamics, so the only way to deal with it is by feedback, treating such an effect as disturbances or process variability.

In this paper, we address the above issues in an industrial case from the food-processing industry, where products pass through a chain of batch and continuous processes. These processes and operations must be synchronized, also with the frequencies of supply arrivals and product demands, to prevent the appearance of bottlenecks and/or waste. In these industries, production starts with preprocessing the raw food ingredients (e.g., thawing, mixing, etc.). Once settled, the foodstuff is packaged in containers from a range of commercial formats and sealed. These can be considered continuous processing lines. Then, before entering the palletizing lines (also continuous), food items are gathered in carts and subjected to thermal treatments (e.g., pasteurization or sterilization) to ensure microbiological safety. These treatments obey pre-established time–temperature profiles, typically dependent on the food type, size, and container geometry.^19^ Indeed, thermal processing constitutes the main bottleneck and the most energy-consuming operation of these facilities, where the energy source (usually in the form of live steam) is shared among redundant thermal process units that operate under different time–temperature profiles.^20^

From a systems point of view, the described problem consists of the coordination of batch concurrent processes (whose duration may vary depending on the shared resources availability) that take place in between continuous production lines. Moreover, the logistics at the interface between continuous lines and the batch processes may also represent a constraint if it is not automated but conducted by a limited number of plant operators in charge of manually moving carts, for instance. This means that the scheduling solution also needs to consider the human in the loop once implemented.^21^

This paper proposes a predefined-precedence scheduling formulation that can run in a closed loop to respond to disturbances at the MES level of the control hierarchy. The proposed MILP approach explicitly models the varying durations of the batch processes that are derived from concurrent resource consumption. In this way, our proposal does not only account for bounds on resource consumption but it incorporates (i.e., predicts to some extent) the effect that future scheduling decisions will have on the process dynamics. Moreover, the developed decision support solution (DSS) considers the necessary human intervention to execute the production schedule.

The rest of the paper is organized as follows: Section II gives the details on the industrial case study on tuna canning; Section III presents the proposed scheduling formulation and the proposed strategy to deal with shared resources; some relevant details on the implementation are given in Section IV; a summary of the many validation tests is presented in Section V; finally, the paper ends with remarks and highlights on actual implementation.

## II Case Study: Tuna Cannery

The proposed scheduling solution was developed for a cannery located in the northwest of Spain. Production starts with deep-frozen tuna arriving at the plant. The amount of ‘raw’ food required to fulfill the work orders programmed by the Enterprise Resource Planning (ERP) system must be first thawed. To do so, frozen tuna is placed in special rooms with controlled humidity and temperature.

After thawing, the foodstuff is cleaned manually. If the whole fish is processed, cleaning includes the separation of different parts such as loins, tuna bellies (typically more appreciated than loin), or tuna crumbs, which will lead to a range of final products. Tuna foodstuff is then manually packaged in metal containers (cans). Then, cans are filled with a preservative liquid such as brine, olive oil, or pickled sauce and sealed in automated lines. The different possible combinations of container formats, filling liquids, and types of tuna foodstuff result in a large number of commercial products.

Following the sealing, compatible food items (similar tuna type and can formats) are gathered in carts that must be subject to a batch thermal treatment known as sterilization, whose primary aim is to kill or inactivate spores or microorganisms, to prevent the formation of harmful compounds such as histamine. Sterilization here is performed in industrial retorts, each consisting of a metallic cylindrical carcass with hermetic doors at both sides in which heating and cooling take place using superheated and cooling water, respectively. A plate heat exchanger is used to heat and cool the water entering each of the retorts.

Note importantly that, once tuna has been thawed, it has to be sterilized within a maximum time window for food-safety reasons. Therefore, production lines must avert delays or blocks to fulfill this first critical-time constraint. Furthermore, in this plant, carts are manually loaded into retorts by plant operators.

**Figure 1:**
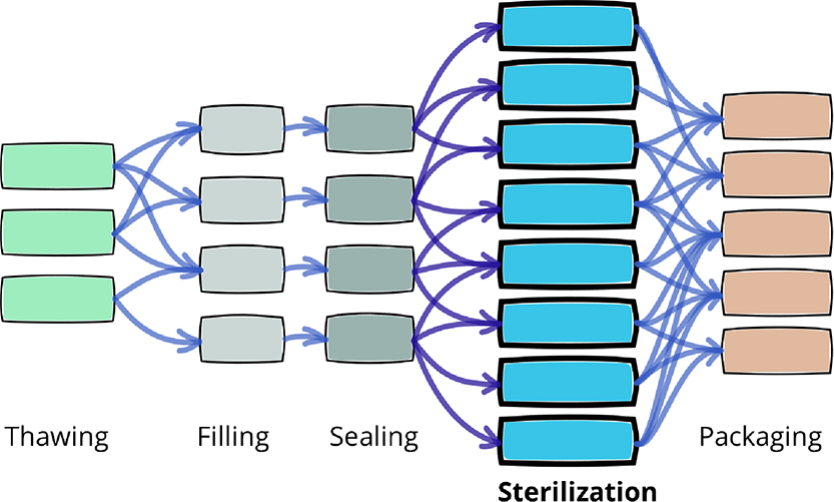
Schema of the main processing stages in a typical tuna cannery. Sterilization is a batch process, usually the main production bottleneck.

After sterilization, carts are unloaded also by the operators into the packaging lines, which wrap the cans in groups depending on the selling format, and they are dispatched to storage. Figure 1 summarizes the process workflow.

The number of carts and the available space for buffering the product before sterilization are limited. This implies that (a) empty carts in the packaging section need to be sent back to the sealing lines by the operators; (b) some paths from sealing lines to retorts are not possible (see Figure 1). Such constraints together with the maximum waiting time of food before sterilization may require launching a thermal process without a retort at full capacity, especially if there is uncertainty on the arrival instant of the next cart filled with compatible food items.

Microbiological safety is typically measured by the so-called microbial lethality.^22^ If the temperature in the food item under sterilization can be measured or inferred from the retort temperature using mathematical models, lethality can be monitored online during the treatment.^23^

In this way, sterilization accomplishes according to pre-established time–temperature profiles (or recipes), typically dependent on the foodstuff (type, size, and geometry), of enough duration so that the lethality achieves a required value. It must be remarked that such thermal treatments induce food-quality losses due to the degradation of nutrients or sensory parameters (e.g., color or texture), which suggests optimizing the temperature-profile shape in a multiobjective fashion.^19^

**Figure 2:**
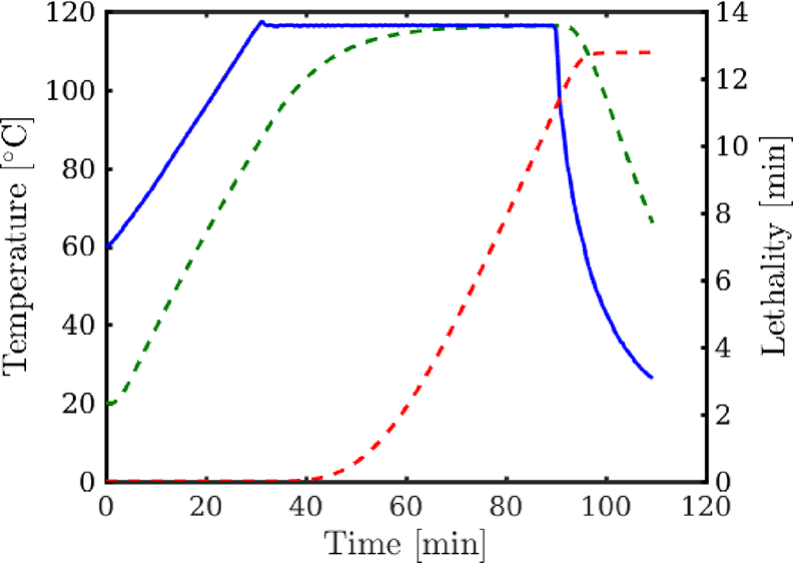
Typical time–temperature sterilization profile. Retort temperature (measured, blue plain line), foodstuff coldest temperature (inferred, dashed green line), and the corresponding microbial lethality (dashed red line).

Figure 2 shows a typical sterilization profile, where three stages are identifiable: (1) the heating or come-up that is the most steam demanding, (2) the plateau where the temperature is kept constant for a given time, and (3) the cooling in which the temperature must go down as fast as possible (avoiding steep pressure drops that might damage the food containers) to avoid excessive-quality degradation and cycle time.

## III Sterilization-Section Scheduling

Retorts are placed in parallel, following the sealing lines. The number of different products to be sterilized in the same retort is limited by the section manager according to (1) compatibility of their thermal treatments and (2) ease in their posterior distribution into the packaging lines by the operators. Sealing lines work simultaneously, producing several products. However, depending on the production demands, different sealing lines can also release the same type of products.

To reduce energy consumption and overall processing time, the operators attempt to fill the retorts with as many carts as possible. Therefore, carts are first gathered in groups up to the retort capacity. Then, such groups must be assigned to one of the closest retorts. If they are busy, the cart groups have to wait in a buffer place near the retort, thus complicating the operator’s movements in a limited space.

Once inside the retort, a temperature controller (usually a PID) is in charge of applying the required sterilization profile (see Figure 2). The steam used to produce superheated water is delivered to the different plate heat exchangers through a unique steam pipe. Therefore, when a sterilization cycle begins, it generates a pressure drop in the pipe. If there are simultaneous pressure drops that the supply cannot quickly compensate for, the steam is trimmed in excess. Henceforth, the retort temperature deviates from the setpoint. This is especially problematic during the come-up stage, where the steam demand is high.

Two ways of action can be followed in such cases:

(a) Typically, the plateau-stage duration is not modified to be sure that the required lethality is reached, with the consequent extension of the whole processing cycle and food quality loss.
(b) Use an advanced predictive control strategy that monitors lethality online and implements a new optimal sterilization profile in case food safety is compromised.^24^

In any case, a plant-wide schedule computed at the upper layer in open loop^25^ may be not valid anymore, and just re-scheduling^26^ may not be feasible if the above control strategies are not integrated in the scheduling algorithm. However, incorporating all details in a plant-wide scheduling formulation results in an extremely complex problem that is not computationally affordable, so aggregated modeling is mandatory in such a case.^25^ Therefore, this work focuses on the online scheduling of the sterilization section, statistically the main bottleneck.

A first simple way to avoid steam-supply issues is to limit the number of sterilizers that can be simultaneously in the come-up stage. However, this approach is very conservative, with a negative impact on productivity. A less conservative enhancement of this idea is to predict the overall steam consumption along the scheduling horizon, computed as the aggregation of several resource-demand profiles, and setting a bound on it.^27^ However, this way increases the number of decision variables and constraints, and some conservatism remains if the resource-availability bound varies but such variation is not known a priori.

Instead of such approaches that assume changes in the sterilization profiles are either negligible or forbidden, the authors propose to integrate the decisions on the operation of the sterilizers with the real-time scheduling to better fulfill the overall planning aims.^28^ In this regard, a sensible idea is to model the changes that overlapping sterilizations may produce in the time–temperature profiles during the come-up stage. This idea is formulated and expanded along the next sections, also providing validation results once the scheduling is implemented onsite in the cannery.

Summarizing, the scheduling of sterilization batches over time involves the following problems:

a) Gathering carts into groups to be sterilized together.
b) Assigning groups to retorts and to synchronize processes.

Different objectives can be sought in the scheduling approach. The most typical are to maximize either product quality or production throughput or to minimize resource consumption. Additionally, the following constraints must be considered: lethality must reach the desired value at the end of each sterilization, and the maximum waiting time between thawing and sterilization must not be surpassed. Furthermore, a smooth synchronization of the sterilization section with the previous and following continuous production lines is needed to prevent the formation of undesired bottlenecks that would significantly affect the production planning given by the ERP. Accordingly, this paper approaches the problem as a closed-loop near-optimal scheduling, which provides support to operators on the decisions of how to gather the carts, which sterilization profile to set in the retorts, and how to re-organize the batch processes depending on the actual plant situation.

### IIIA Scheduling Model

The scheduling model presented below concerns the sterilization section (Figure 1). Product carts delivered by the sealing lines, their corresponding sterilization cycles, retort state, and resource availability are inputs to the proposed DSS.

The scheduling is formulated as a mixed-integer linear programming (MILP) problem. The overall problem can be conceptually split into three sub-problems: carts grouping, retort assignment, and sterilization cycle synchronization.

Let us define the following sets to describe the sterilization section:

represents commercial products.
represents carts to be sterilized. It includes the carts that have already arrived at the section and the forecast of those expected to arrive within a given time horizon.
represents the cart groups that will be sterilized in the same retort. It corresponds with the sterilization slots.
represents retorts.
represents sealing lines that release the full carts.

In addition, , represents the subset of sealing lines whose carts can be loaded inside retort *k*, i.e., the available paths to the retorts from every sealing line.

Known parameters to the problem we have are the following: the expected arrival time of every cart (or the actual one if the cart has already been released from the sealing lines); the type of commercial product loaded in a cart; the production line that filled each cart; and the required sterilization profile for every product. These data are provided by the manufacturing execution system (MES) in the plant. From these data, the following values can be defined:

indicates the time between the current instant and the expected arrival of cart *i* in minutes. It may be negative if the cart has already arrived.
is the time gap since cart *i* arrives at the sterilization section and its deadline to start sterilization due to the potential formation of histamine in the food product.
indicates the product type in each cart. If *C*_*i*, *h*_ = 1, it means that cart *i* has cans of product *h*.
indicates which sealing line releases each cart. If *Q*_*i*, *l*_ = 1, line *l* has released cart *i*.
set the duration of the come-up and cooling stages for all products, respectively.
indicates the duration of the plateau stage for each product. Note that the duration of the come-up and cooling stages (ξ,χ) can also differ between products, but these time differences are both embedded, without loss of generality, in the plateau stage to ease the formulation.
indicates the maximum number of different products that can be included in the same slot. This parameter is included to facilitate the posterior discharge of sterilizers to packaging lines by the operators.
indicates when a busy retort *k* will be released.

### IIIB Gathering and Assigning Carts to Retorts

Carts are grouped in batches of up to Γ carts (maximum size of the retort, in carts number) to be assigned to the retorts. The following decision variables are defined for this purpose:

, which equals 1 if slot *g* includes at least one cart of type *h*.
, which equals 1 if cart *i* belongs to slot *g*.
, which equals 1 if slot *g* is assigned to retort *k*.
. Binary variables that result from the conjunction of the two previous ones, meaning that cart *i* is introduced in retort *k*.
denotes whether slot *g* is used in the schedule (*U_g_* = 1) or not (*U_g_* = 0).
specifies the start instant of the sterilization process for slot *g*.
indicates the duration of the sterilization cycle for slot *g*, i.e., the slot duration.

A continuous-time basis has been chosen to formulate the problem to avoid the excessive granularity of the discrete-time approaches. Hence, the following sets of constraints are defined:
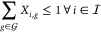
1
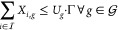
2
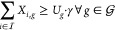
3
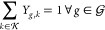
4
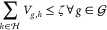
5
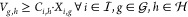
6
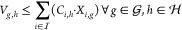
7

Inequality 1 establishes that each cart must be processed only once. Note that all carts in  (i.e., in the forecast horizon) are not forced to belong to a slot, considering that production forecasts may vary and the scheduling algorithm will run periodically in a closed-loop fashion. Inequalities 2 and 3 define the number of carts in the sterilization slots, with the retort capacity (Γ) being the upper bound. The lower bound γ ≥ 1 is an optional parameter selected by the user to prevent quasi-empty batches. Depending on the chosen objective function, such a parameter may or may not be required. Equation 4 ensures that each sterilization slot is assigned only to one retort. Inequality 5 sets the maximum number of products per slot ζ. The convex hull formulation,^29^ with inequalities 6 and 7, is used to model the product references included in each slot.

The logical conjunction between variables *X*_*i*, *g*_ and *Y*_*g*, *k*_ (the relation cart to retort) is linearized by the following inequalities:
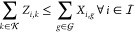
8
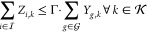
9
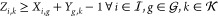
10

Using such *Z*_*i*, *k*_ variables, the paths allowed from production lines to retorts are modeled by
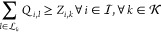
11

Next, some relations describing the time constraints of the assignment will be presented. The starting time of every sterilization cycle is bounded by the carts included in its slot *g*, i.e., it has to start before the food product in the cart with the shorter waiting-time gap θ_*i*_ in the group *g* is unsafe, but never before all carts to be grouped in such slot have been released from the sealing lines. This is modeled by the following inequalities:
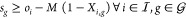
12
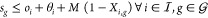
13where *M* is a user-defined (big enough) number according to the *big-M* approach,^30^ used to relax the constraints (i.e., deactivate) when *X*_*i*, *g*_ = 0.

Note, however, that the above inequalities only consider the carts chosen to be sterilized, not those left waiting for the next run, i.e., . So far, no constraint has forced the carts to be included in any sterilization process. To prevent carts that necessarily have to start sterilization before the next run from being left out of the retorts, we add the following:
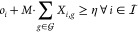
14where η is the robustness horizon timestamp, meaning that only the carts that arrive before η (i.e., *o_i_* < η) must be included in a slot. Timestamp η is defined relative to the current time and will increase as time does so. Thus, new carts will be constrained by inequality 14 in every execution of the scheduling algorithm.

Moreover, those carts that are already waiting for being sterilized in a particular retort should not vary their assignation (note that carts grouping and displacement are performed by operators). To ensure this, let us define the subset  formed only by those carts waiting and already assigned to a retort by a previous algorithm run. Hence, the following constraint is included in the model:
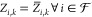
15where *Z̅*_*i*, *k*_ are the cart-to-retort assignations recovered from the solution of the previous run.

*Remark 1.* This formulation releases extra degrees of freedom for better reaction to unexpected future disturbances and increases the goodness of the solution in a closed-loop fashion. However, consequent with the discrete-time control theory on sampling, the optimization must be run at periods much lower than the robustness horizon to avoid infeasibilities (i.e., instability in closed-loop control systems).

Temporal order of the slots *g* has to be defined. A general-precedence approach is often presented in the scheduling literature for such an aim. This approach declares extra binary variables that determine whether two processes allocated to the same equipment precede each other. However, apart from increasing the problem size, it is tedious to synchronize the whole set of slots following such formulations. To avoid such issues, the authors propose a strategy with preordered slots (predefined precedence). In this way, the slots are neither assigned previously to any equipment nor to any contents. Henceforth, as we ensure that every sterilization process will be only executed once, these will happen in order. The strategy is as follows: First, we define the set  as an ordered one. Then, we extend this order to the slots’ starting time and we use it to organize the works that have been assigned to the same unit. Formally, this is enforced via
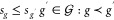
16
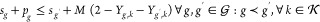
17

Finally, note that some retorts can have ongoing sterilization at each optimization run. We define  as the time instant at which a busy retort becomes available to start loading carts again: It will be zero for an empty retort; otherwise, it will be set to the remaining duration of the sterilization cycle plus the time required to remove the carts from the retort (value known in advance). Therefore, the start time of each slot assigned to a retort cannot be before the end time of the previous slot in such a retort:
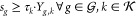
18

### IIIC Startup Synchronization under Shared Resources

The steam to heat the water in plate heat exchangers is a limited shared resource and its availability affects the sterilization dynamics. Consequently, the effects of simultaneous retort operation need to be considered explicitly in the scheduling formulation.

The highest steam demand happens during the come-up stage, so if some retorts are simultaneously in such a critical stage, the overall demand often surpasses the capacity of the steam supply. The consequence is that, for the involved slots, the time required to reach the temperature set point of the plateau stage increases with the steam-pressure drop. To include this effect in the problem formulation, the duration of the come-up stage (*t*_*h*_) of a particular slot is assumed to increase proportionally to the number of other slots whose come-up stages overlap at some time  This approximation appeared to be close to reality in this case study, according to the plant historian and expert personnel. Hence, *t_h_* gets a minimum value of ξ (duration of heating when there is no pressure drop in the supply), plus a variable value defined by the proportionality constant ξ_p_

**Figure 3:**
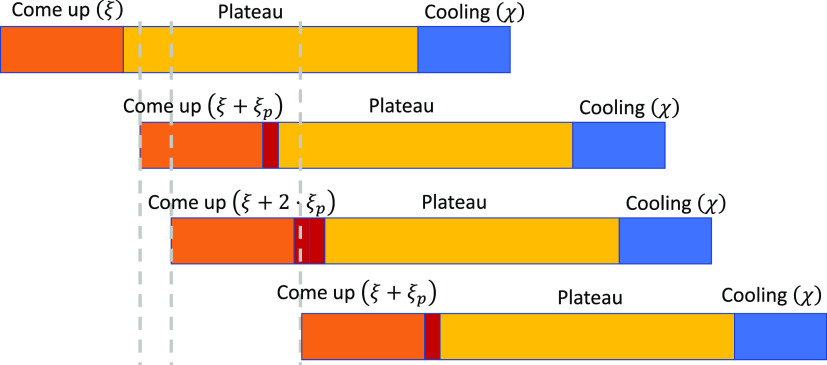
Example of the influence of simultaneous resource consumption among the slot’s duration. Red bars show the varying extensions of the come-up stages.

Figure 3 shows different overlapping situations of four retorts that might appear. Each slot is represented by a bar with red, yellow, and blue colors corresponding to the come-up, plateau, and cooling stages. Heating in the first slot (*g*_1_) does not coincide with heating of any other slot; therefore, the come-up time remains as ξ; meanwhile, the heating of the second and fourth slots (*g*_2_ and *g*_4_) are executed simultaneously to *g*_3_, ergo their times are increased by ξ_*p*_ each; finally, the third slot (*g*_3_) coincides with both *g*_2_ and *g*_4_, so its come-up increases by 2ξ_*p*_.

According to this approach, the heating time ξ (fixed parameter in the formulation of Section III-A) is modified to become a new variable, . Then, the relation among the come-up stages of near slots is modeled by:
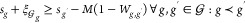
19
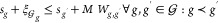
20where  are new binary variables that determine whether the come-up stages of slots *g* and *g*′ coincide. If *W*_*g*, *g*^′^_ = 1, slot *g*^′^ will start before the come-up of slot *g* has ended, eq 19. Otherwise, the come-up stages will not coincide in time and the steam availability will not be affected, eq 20. In order to trim redundant options, hence lowering the computational burden, the following inequalities are added to constrain *W*:
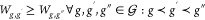
21
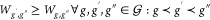
22

This forces that, if the come-up stages of slots *g* and *g*′′ in the ordered set  coincide, all the in-between slots *g*′ must coincide too. Once the relation among slots is modeled, the new heating times are computed for all  by
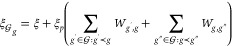
23

Actually, these constraints have to be set only for the  next slots, which are the ones that can be influenced by the current retort. Note that the maximum number of sterilizations starting at the same time is the number of retorts. This reduction is also applied to binary variables *W*_*g*, *g*^′^_.

*Remark 2.* In the case that such an effect of processing-time increment due to concurrent-steam consumption is nonlinear but can be represented by a convex relationship, a piecewise linear approximation of desired accuracy can be set up via the intersection of *N* inequalities *t_h_* ≥ ξ + *n_h_* ξ_*i*_ + β_*i*_, *i* = 1, ..., *N*, in order to preserve the model linearity. A combination of such constraints with the minimization of the objective function (e.g., the makespan) would provide a tight solution, analogous to what was proposed by the authors^28^ to integrate scheduling and control via Pareto frontiers.

Finally, the duration of the sterilization slots is constrained by eq 23 and the following consideration. Every product  is associated with an optimal plateau duration *r_h_*. However, sterilizations can be extended up to a maximum span δ with a sensible loss in the product quality properties.^19^ This feature lets merging up to ζ products, as constrained by eq 5. Hence, slot durations are not fixed but bounded by
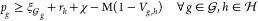
24
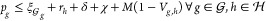
25

Note from eq 24 that the allowed variation is only acceptable in a positive sense.

*Remark 3.* The reader may realize that this modeling approach overestimates the actual duration of the come-up stages, as the slot durations are equally affected if they coincide at the beginning of the heating or just in the last few minutes. To reduce this conservatism, the come-up stage could be split into divisions, each associated with a different proportionality constant ξ_*p*_ in eq 23 and to include as many binary variables *W* as divisions made to mark where the slots coincide. Details are omitted for brevity.

### IIID Objective Function

The proposed scheduling model guarantees that all cans will be sterilized with an adequate thermal treatment within a time window shorter than the robustness horizon, η. The objective function to optimize is usually economic, although this does not have to be always the case in scheduling.

A typical objective is to minimize the makespan. In this case, the scheduling problem would look like
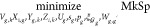

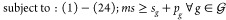
26where  stands for the makespan. However, if the workload of the sterilization section is huge, a more sensible objective would be maximizing productivity, possibly at the price of reduced quality or higher resource consumption. For instance,  will increase the number of carts per sterilization cycle. In this case, it is recommended to constrain the makespan, e.g., 

In periods of lower production demand, product quality, energy usage, or resource consumption could become the objective to optimize, for instance, by lowering the temperature setpoint of the plateau stage at the price of longer sterilization times *r_h_*.^19^

## IV Implementation through the MES

An effective implementation of the proposed closed-loop scheduling approach needs to be integrated with the plant MES. Moreover, as cart displacement is a task manually done by the section operators, a suitable decision-support system (DSS) needs to be provided to consider human intervention.^17^

Concerning mathematical optimization, the above-proposed model was coded in Julia^31^ using libraries that include an OPL (Optimisation Programming Language) translator for easing the definition of multiple constraints over sets. Such is the case of the JuMP library,^32^ which lets the programmer code directly using mathematical programming formulations on Julia. This approach increases the communication capacity of the MILP optimization with external software and databases. As solvers for MILP optimization, two commercial ones have been used, CPLEX 12.9 and Gurobi 8.1, both reporting similar performance in this problem (details in Figure 9a).

The scheduling optimization is executed every 15 min. Since the sterilization processes take much longer (from 60 to 190 min depending on the product), this can be considered real-time execution. Nonetheless, the plant state is continuously updated in the MES.

**Figure 4:**
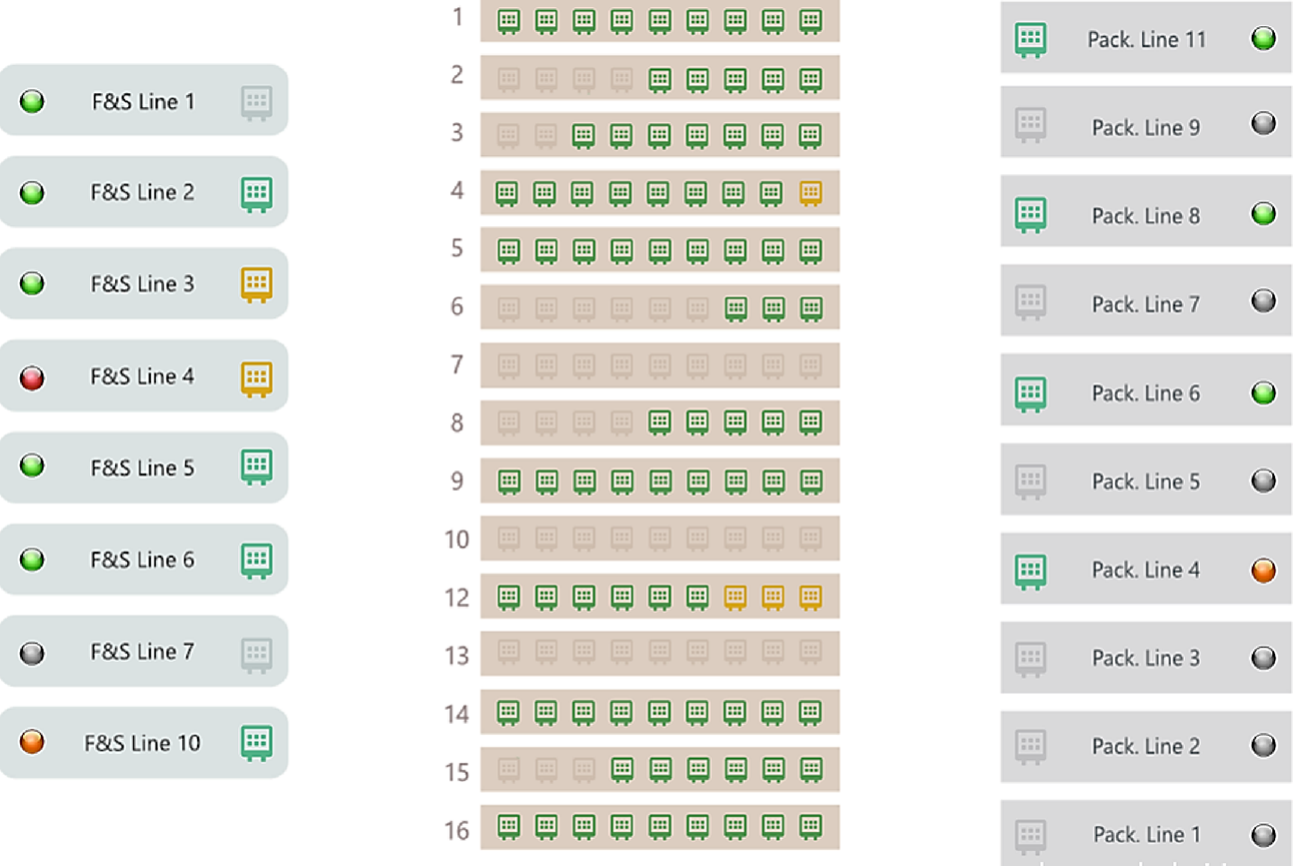
Overview of the state of the sterilization section in real time.

**Figure 5:**
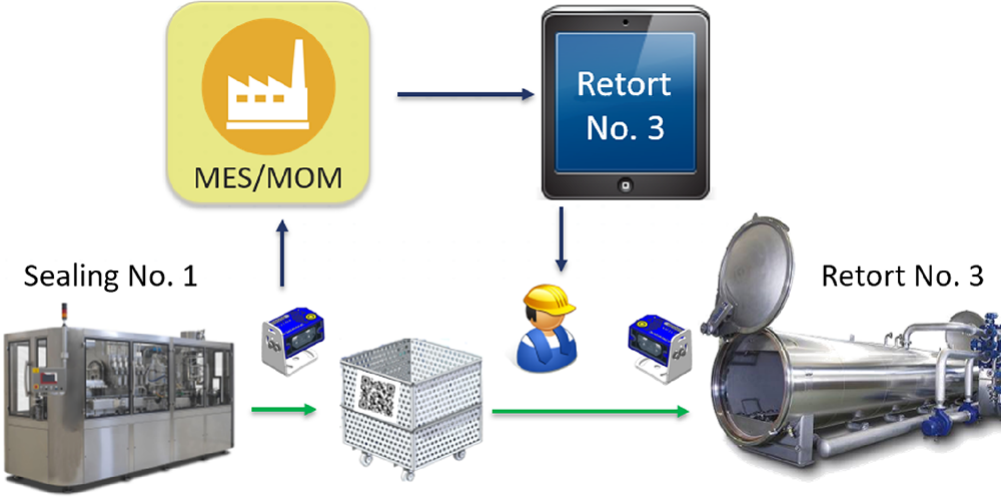
Example of operator workflow with the DSS. Each cart is monitored by the MES via QR code scanners located in the equipment.

To gather cart data and to build the plant-state information required for running the optimization, each cart is identified by a QR code that is read by scanners located at the output of each filling and sealing line as well as at the retort entrance and exit sides. In this way, a compact monitoring system like the overview displayed in Figure 4 is available. To close the loop with the operators in charge of cart logistics, the computed optimal schedule and the path information are available to operators through a QR code placed in each cart (Figure 5). Note that successive runs of the scheduling algorithm do not change the cart-retort assignment for those carts that already arrived at the sterilization section and have been assigned to a retort, see model constraints 15. This provides consistent suggestions to the operators from run to run.

**Figure 6:**
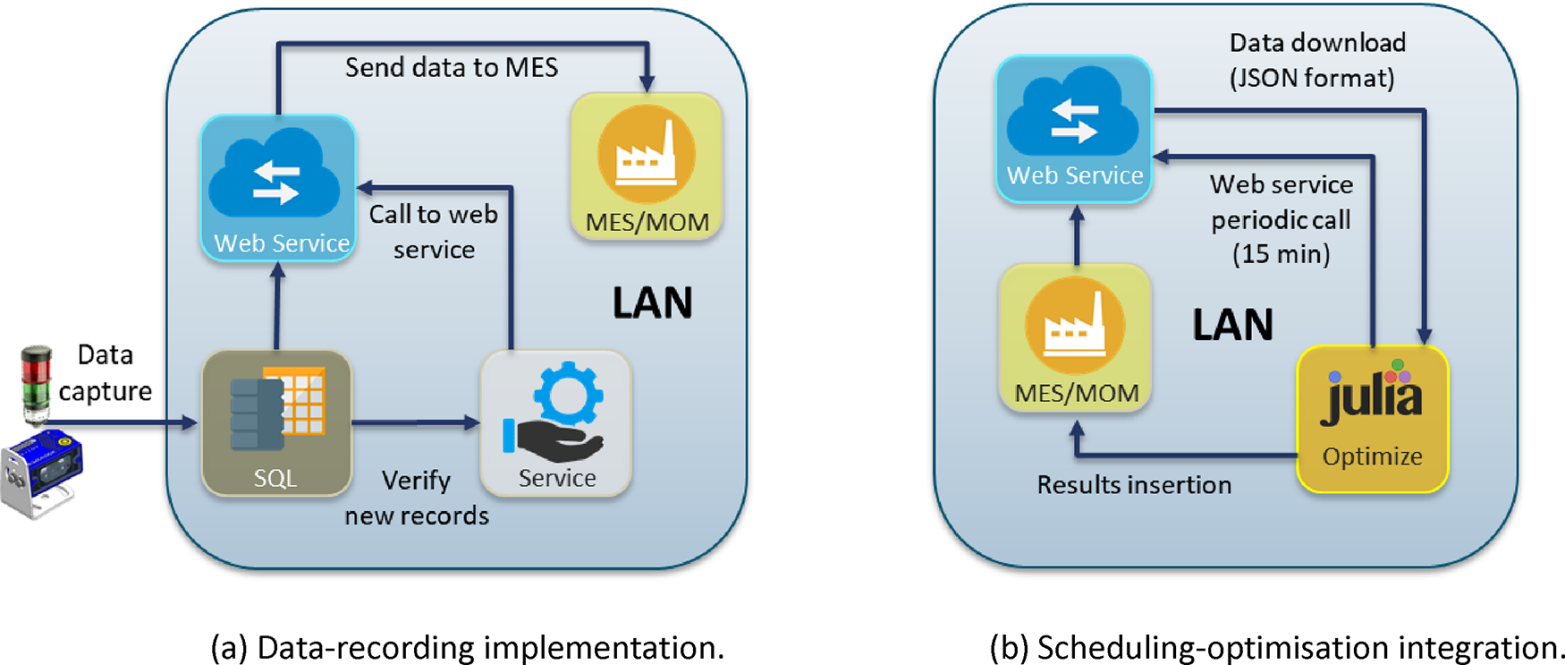
Infrastructure developed to implement the closed-loop scheduling.

Figure 6 depicts the software implementation, where the data recording and the periodic run of the scheduling optimization are performed via web services in the local area network of the factory. Plant data acquired by the QR scanners is stored in a database (SQL for instance) and new updates call the web service, which is in charge of sending the data to the MES system (Figure 6a). In this way, the current plant state is available through the MES. The scheduling optimization routine in Julia runs every 15 min. Each run calls the web service to get the updated data from the MES and sends back the newly computed schedules once the solution is available, see Figure 6b.

### IVA System Evaluation

This section presents a concise overview of the many evaluation tests performed with actual plant data an onsite after implementation.

The setup is as follows. The optimization problem (26) runs in a moving-horizon fashion with a period of 15 min. The robustness horizon defining timestamp η is 120 min. These values have been selected after testing different setups and discussing the obtained solutions with the plant engineers. The considerations were (a) being able to compute sensible solutions in about a minute, (b) capturing the system dynamics, and (c) having a good tradeoff between long scheduling horizons and flexibility to react against unplanned events. The sterilization section has 16 retorts () and receives carts from 10 different sealing lines (). Each retort accepts carts coming only from the closest five sealing lines. There are 12 different sterilization profiles (pairs of time *r_h_* and temperature set point), although the number of product commercial references is much larger. After normalizing the profiles (remember that time differences in the come-up and cooling stages are embedded in *r_h_* with no loss of generality), the standard duration of the come-up stage is set to ξ = 15 min and the cooling stage is of χ_*h*_ = 10 min. The proportionality constant defining the extra increase due to concurrent steam consumption is estimated to be ξ_*p*_ = 5 min/slot. The carts included in the problem, set , are the ones arriving or planned to arrive in less than 3 h, and the group sizes can vary between γ = 1 and Γ = 9 carts. Each group cannot include more than ζ = 3 different products. The maximum difference among the reference sterilization durations of the different products gathered in the same group is constrained to δ = 5 min, in order to avoid excessive quality degradation.

**Figure 7:**
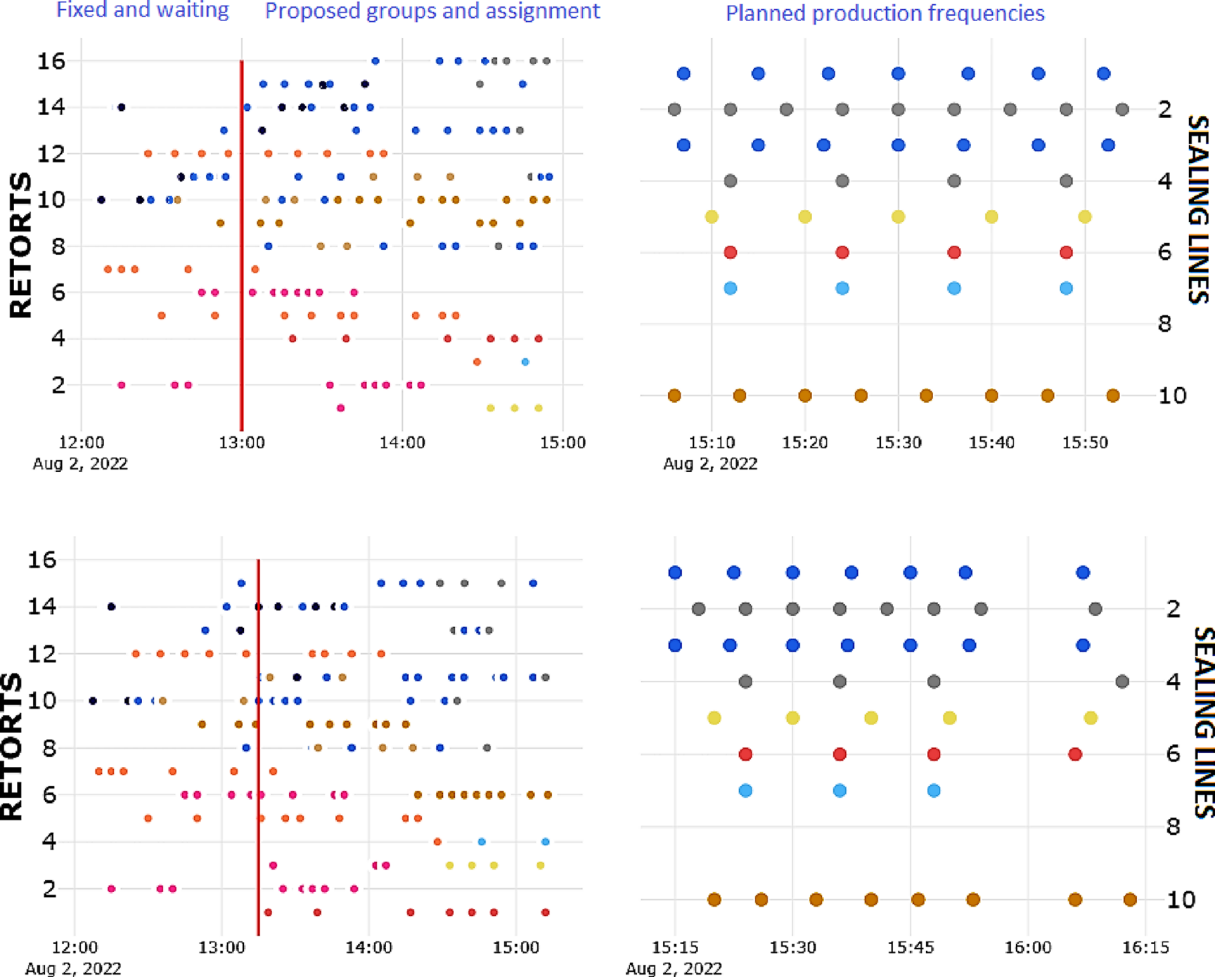
Carts gathering and assignment (left-side charts) given the current plant state and expected carts production at two consecutive optimization runs. Carts expected to arrive within the robustness horizon (2 h) from the current time (red vertical line) are grouped, while those expected to be produced further in the sealing lines (right-side charts) will be progressively included in the next runs. Dot color represents the different product commercial references.

Figure 7 shows the proposed carts gathering and assignment to retorts at two consecutive runs. In these charts, time evolution is represented in the horizontal axis whereas the vertical axis represents either retorts (left charts) or sealing lines (right charts). The current time instant is marked by a red vertical line. Dots represent the timestamp when a cart filled with some product (type distinguished by the dot color) either arrived or is expected to arrive at the sterilization section. Note that those carts that already arrived do not vary their assignment from the first run to the second one, while those that have not yet arrived may change. The picture is analogous to the one generated by Model Predictive Control, which distinguishes the actions that are already committed (left from the red line in Figure 7) from those that are computed to be applied at the current time and in the future. The future ones are being recomputed in the next execution period.

**Figure 8:**
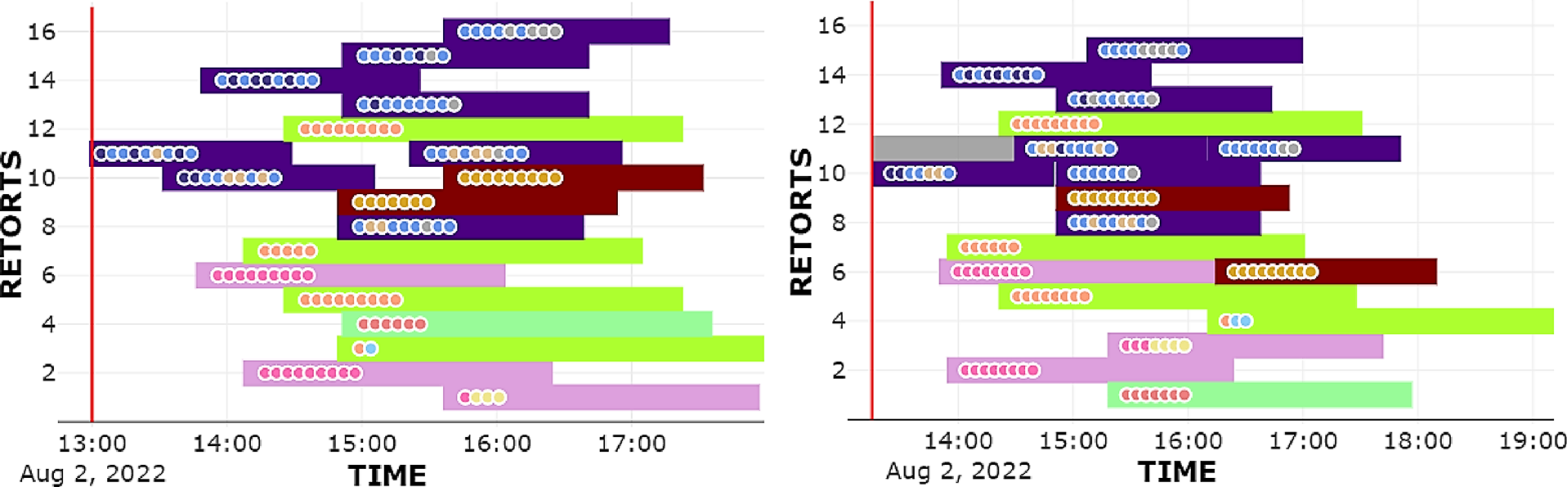
Scheduling corresponding to the algorithm runs at the time instants of Figure 7. Bar colors represent different types of sterilization profiles.

The resulting schedules were analyzed by the plant engineers using Gantt charts for results verification before implementing the solution onsite. The Gantt charts corresponding to the runs in Figure 7 are depicted in Figure 8. In there, sterilization cycles, or slots , are drawn as colored bars whose horizontal length corresponds with their time duration. The same bar color means equivalent thermal treatments. Note, however, that the bar lengths (time duration) can vary even for slots of the same color, due to the come-up extensions  in the case of concurrent steam consumption.

If a retort is already busy when the optimization is run (due to a previous sterilization cycle that has not yet finished), it is represented by a light gray rectangle that extends up to the expected end instant of that slot (retort no. 11).

It can be noted in Figure 8 the differences between two optimal schedules computed consecutively. This may be wrongly associated with excessive solution nervousness, an undesired property in many scheduling applications. However, it is desired here: a schedule may be optimal for the carts planned to arrive within 3 h, but there may be a better reallocation after 15 min considering new arrivals. Moreover, there is always uncertainty in the actual arrivals (delays due to machine or operator issues), so the scheduling needs to be flexible. Evidently, batches that have already started the sterilization are forced to remain invariant.

Data gathered after the DSS was onsite show a 5% improvement in the utilization factor of the sterilizers and reductions of about 1% in the steam consumption and 1.5% in the water consumption. Note, however, that a fair comparison is difficult to quantify, as scheduling decisions made by operators before the DSS implementation were in situations that are likely not equal to those of the few tests with the DSS onsite.

### IVB Handling Infeasibilities

Despite the optimal scheduling of the sterilization section, bottlenecks can still arise due to equipment breakdowns, operators incurring extra delays when releasing filled carts and arranging the empty ones, lack of resource availability, or simply because the production frequencies of the upstream lines are too high. As a consequence, the maximum waiting time constraint can be violated for some carts, so scheduling becomes infeasible. Moreover, this poses a safety risk due to the potential formation of histamine inside the food cans.

In this context, carts that are predicted to exceed the maximum waiting time can be distinguished into two classes: on the one hand, the ones that are being filled or already waiting in the buffer to be sterilized and, on the other hand, those that do not yet exist but are expected to be produced in a near future.

From the algorithmic side, such infeasibilities are avoided by adding extra slack variables  to relax the right-hand side of eq. 13 and including them in the objective function with a strong penalty to force their minimization. In practice, however, this does not solve the problem for the carts already waiting as well as for those being filled, which will exceed their waiting time anyway. In these cases, they will be sterilized extending the time (at the price of quality reduction) and put in quarantine afterward for analyzing the presence of histamine.

Nevertheless, predicting such infeasible situations through the scheduling algorithm is useful for the carts that are planned but do not exist yet. Hence, what is sensible is to use this feedback to decrease the production rate in upstream sections, preventing thus the future bottleneck and its harmful consequences. Figure 7 shows an example of such an action, where the expected production by the sealing lines at instant 16:00 h is modified from the first run to the next one.

### IVC Computational Evaluation

For the above problem instances, the problem size with the proposed predefined-precedence approach was 136,584 constraints, 76 real variables, and 5030 binary ones. The elapsed CPU time to solve the optimization up to 0% optimality gap was less than 30 s, except in a few atypical situations. However, following a general-precedence formulation, no feasible solution is obtained after 10 min of CPU time, although the problem size barely increases (153,336 constraints, 76 real variables, and 5655 binary ones).

**Figure 9:**
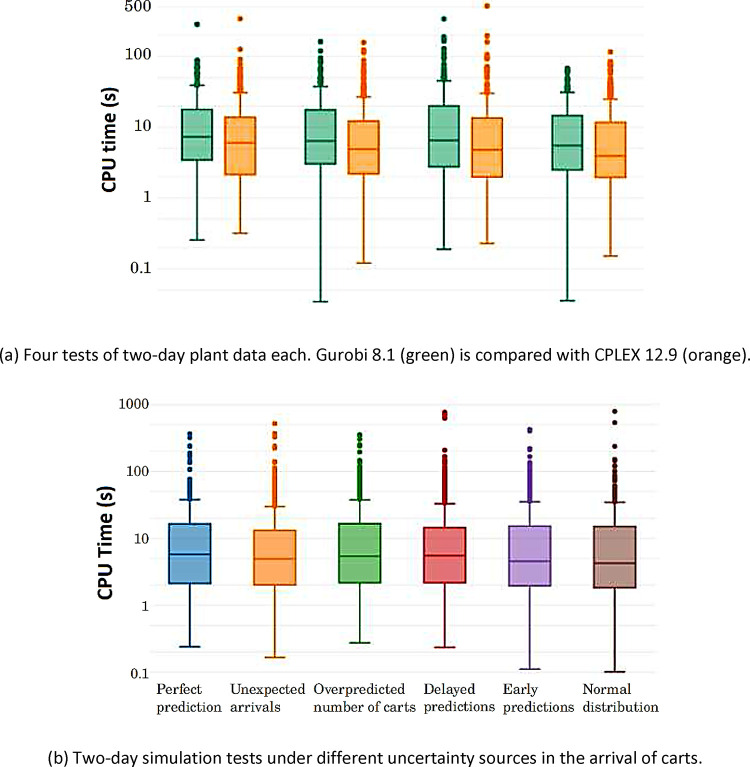
Distribution of the observed CPU times with the proposed closed-loop scheduling approach. Upper dots are scarce atypical values.

Results from the first tests onsite, summarized in the box plots of Figure 9a, show that the expected CPU time to solve the scheduling optimization with the setup proposed in this paper is less than 10 s. This was also confirmed in silico, by testing the algorithm under different sources of uncertainty in the carts-arrival predictions, see Figure 9b. The numbers in these figures have been obtained in an AMD Quad Core R5-2500U CPU laptop, enabling multithread execution in both CPLEX and Gurobi solvers.

## V Conclusions

This work shows an online scheduling solution for the sterilization section of a tuna cannery, where parallel batch units operate asynchronously but consume limited shared resources. The proposed approach deals with the existing uncertainty through feedback, in a closed-loop implementation, that was successfully integrated into the factory MES. The first numbers after onsite evaluation show a 5% improvement in the equipment utilization factor and a 1% reduction in the steam consumption. Thus, the presented approach stresses the importance of implementing short-term scheduling in a closed loop for being able to react to changes in production with lower response times, reducing the formation of bottlenecks and improving resource efficiency. Note also that long-term open-loop planning and scheduling are also relevant in the closed-loop scheme, as a source of estimated items arriving in the future to the considered production section.

An efficient approach when developing a model for closed-loop scheduling is critical to provide solutions in real time. Nevertheless, through this experience, we want to highlight that there is no ‘magic’ approach that best fits all cases: learning accurate enough AI models was inviable in this industrial case study due to the extremely large action-state search space that plant data needs to cover. However, the formulation of MILP models for systems sharing limited resources in a batch-continuous arrangement is also complex to derive: although discrete-time modeling approaches become the natural formulation, they are just an approximation of the shared-resource constraints that may be not computationally suitable for real-time setups in realistic problems, especially when the prediction horizon and the online re-scheduling requirements are in different time scales.

The proposal in this work follows an alternative continuous-time formulation based on predefined precedence slots to deal with such a complex scheduling problem in the presence of uncertainty and limited shared resources, consumed by batch units at non-constant rates over the batch time. The proposed solution also integrates batch and continuous production lines to effectively execute a given production plan. The adopted mathematical formulation provides solutions in times short enough to be implemented in real time.

Nevertheless, it is worth remarking that fully closing the loop at the scheduling/planning level, replacing personnel by an automatic scheduling system, is still a utopia in many industrial facilities not yet fully automated, or whose layout was defined following former criteria. Consequently, our solution integrates the human intervention to close the loop by assisting operators in compliance with the execution schedule, which improves reliability (reducing human errors) and acceptability in the implementation phase.

This paper presented a solution customized for tuna canneries, but the formalism can be easily tailored to other food-production facilities (packaged food and ready-to-eat products). Furthermore, the approach could be extrapolated as well to different sectors that share similar features to this case study, for instance, in the consumer-goods industry, chemical facilities, or tile factories, by considering some units as sources rather than consumers in the formulation and adapting the time constraints to the particular features in each case. However, for the proposed approach to be correctly integrated with the overall plant planning/scheduling, it needs to be applied to a critical or bottleneck section. Otherwise, optimally scheduling just a part of the process may hamper production in downstream processes, even leading to infeasibilities by constraint violation. The formulation may be also either conservative or leading to infeasible schedules in practice if the effect of concurrent-resource consumption on the equipment dynamics (e.g., batch-time extensions in eq 23) cannot be reasonably well-approximated by a piecewise linear relationship.
